# Semi-deconvolution of bulk and single-cell RNA-seq data with application to metastatic progression in breast cancer

**DOI:** 10.1093/bioinformatics/btac262

**Published:** 2022-06-27

**Authors:** Haoyun Lei, Xiaoyan A Guo, Yifeng Tao, Kai Ding, Xuecong Fu, Steffi Oesterreich, Adrian V Lee, Russell Schwartz

**Affiliations:** Computational Biology Department, Carnegie Mellon University, Pittsburgh, PA 15213, USA; Computational Biology Department, Carnegie Mellon University, Pittsburgh, PA 15213, USA; Department of Biological Sciences, Carnegie Mellon University, Pittsburgh, PA 15213, USA; Computational Biology Department, Carnegie Mellon University, Pittsburgh, PA 15213, USA; Department of Pharmacology and Chemical Biology, UPMC Hillman Cancer Center, Magee-Womens Research Institute, Pittsburgh, PA 15213, USA; Department of Biological Sciences, Carnegie Mellon University, Pittsburgh, PA 15213, USA; Department of Pharmacology and Chemical Biology, UPMC Hillman Cancer Center, Magee-Womens Research Institute, Pittsburgh, PA 15213, USA; Department of Pharmacology and Chemical Biology, UPMC Hillman Cancer Center, Magee-Womens Research Institute, Pittsburgh, PA 15213, USA; Computational Biology Department, Carnegie Mellon University, Pittsburgh, PA 15213, USA; Department of Biological Sciences, Carnegie Mellon University, Pittsburgh, PA 15213, USA

## Abstract

**Motivation:**

Identifying cell types and their abundances and how these evolve during tumor progression is critical to understanding the mechanisms of metastasis and identifying predictors of metastatic potential that can guide the development of new diagnostics or therapeutics. Single-cell RNA sequencing (scRNA-seq) has been especially promising in resolving heterogeneity of expression programs at the single-cell level, but is not always feasible, e.g. for large cohort studies or longitudinal analysis of archived samples. In such cases, clonal subpopulations may still be inferred via genomic deconvolution, but deconvolution methods have limited ability to resolve fine clonal structure and may require reference cell type profiles that are missing or imprecise. Prior methods can eliminate the need for reference profiles but show unstable performance when few bulk samples are available.

**Results:**

In this work, we develop a new method using reference scRNA-seq to interpret sample collections for which only bulk RNA-seq is available for some samples, e.g. clonally resolving archived primary tissues using scRNA-seq from metastases. By integrating such information in a Quadratic Programming framework, our method can recover more accurate cell types and corresponding cell type abundances in bulk samples. Application to a breast tumor bone metastases dataset confirms the power of scRNA-seq data to improve cell type inference and quantification in same-patient bulk samples.

**Availability and implementation:**

Source code is available on Github at https://github.com/CMUSchwartzLab/RADs.

## 1 Introduction

Computational methods for resolving single-cell clonal evolutionary dynamics (Greaves and Maley, 2012) have become a central part of modern cancer genomics research as ever more powerful genomic tools have become available and as the role of tumor heterogeneity and clonal evolution in cancer progression have become more apparent (Beerenwinkel *et al.*, 2016). The fundamental goal of such methods is to characterize the genetics and genomics of tumor cells and various other cell types infiltrating them and understand how these populations of cells predict future tumor progression and evolve over its course. Numerous variations on this basic framework have been developed, for different kinds of genomic data (e.g. DNA-seq versus RNA-seq, bulk versus single-cell) or different research questions (e.g. understanding genetic versus phenotypic evolution) (cf., Schwartz and Schäffer, 2017). In the present work, we focus on one specific scenario: understanding RNA evolution in settings in which bulk and single-cell data are available for different time points, samples or stages of progression.

Prior to the emergence of practical single-cell methods, most techniques for reconstructing clonal evolution depended on *genomic deconvolution* (Lu *et al.*, 2003), in which one seeks to resolve clonal evolution by computationally inferring activities of homogeneous cell populations from mixtures of genomic data contained in bulk samples (Schwartz and Shackney, 2010). Methods for this problem can roughly be classified into two classes: partial deconvolutional algorithms, which interpret data in terms of a reference matrix of known cell types, and complete deconvolutional algorithms, which infer cell types *de novo* by comparison of multiple samples. Examples of partial deconvolution methods include CIBERSORT, which makes use of a pre-defined LM22 profile matrix and CIBERSORTx (Newman *et al.*, 2019), which derives a profile matrix for interpreting bulk data using reference single-cell data. See Avila Cobos *et al.* (2020) for a comparison of different partial deconvolution algorithms and related data transformation methods. Examples of complete deconvolution methods include Geometric Unmixing (Schwartz and Shackney, 2010), which proposed an archetype analysis method based on geometries of genomic point clouds; DSA (Zhong *et al.*, 2013), which treats deconvolution as a matrix factorization problem; LinSeed (Zaitsev *et al.*, 2019), which identifies a set of anchor genes through linear correlation and uses DSA to solve for the non-anchor genes; NND (Tao *et al.*, 2020a), which poses partial deconvolution problem as a matrix factorization to be solved with gradient descent implemented through a neural network; and RAD (Tao *et al.*, 2020b), which solve s the formulation of NND using a hybrid optimizer with improved accuracy and speed.

Single-cell genomics has rapidly displaced deconvolutional methods for tumor genomic analysis, particularly for RNA-seq variants, as single-cell sequencing has become reliable and cost-effective (cf. Kuipers *et al.*, 2017; Lim *et al.*, 2020). Although single-cell sequencing introduces its own computational complications and data quality issues, having large numbers of direct single-cell measurements leads to substantially greater resolution for single-cell variation than is possible for deconvolutional methods even with high quality bulk data. Nonetheless, deconvolutional methods remain necessary in practice for a variety of real-world use cases. Single-cell data is not typically possible for older archived samples and the field still lacks large cohorts of single-cell tumor genomic data comparable to bulk resources such as the influential Cancer Genome Atlas (Chang *et al.*, 2013) or International Cancer Genome Consortium (Zhang *et al.*, 2011) datasets. The problem is particularly acute for current studies of patients being tracked longitudinally, e.g. in using clonal phylogenetics to understand metastatic progression (Naxerova and Jain, 2015). Patients being seen today for metastatic disease may have been first diagnosed years earlier and characterizing their tumors’ evolution can require comparing recent samples for which single-cell data is practical with archived samples for which only bulk data is possible.

This work was developed specifically to address scenarios such as this, in which one seeks to understand longitudinal progression of a cancer by comparing samples some of which may be amenable to single-cell methods (e.g. recent metastases) and others that can only be examined by bulk methods (e.g. an archived primary tumor biopsy). It accomplishes this by developing a hybrid algorithm to infer genomics and clonal frequencies in both bulk and single-cell samples, using single-cell data in some samples as partial references for bulk data in others. In this regard, it follows a strategy of mixed bulk and single-cell data previously applied in tumor evolutionary studies primarily with single-cell and bulk DNA-seq (Lei *et al.*, 2020; Malikic *et al.*, 2019a,b; Salehi *et al.*, 2017) or using bulk DNA-seq to guide interpretation of single-cell RNA-seq data (McCarthy *et al.*, 2020; Shafighi *et al.*, 2021). Bulk and single-cell RNA-seq has been previously combined in bMIND (Wang *et al.*, 2021), although with a different goal of using paired data from single samples to better reconstruct cell type profiles. Reference single-cell RNA-seq has also been used to train neural networks to deconvolve independent bulk RNA-seq data (Menden *et al.*, 2020). Our method poses the problem using a matrix factorization formulation comparable to earlier bulk deconvolution methods, drawing on ideas from hybrid bulk/single-cell DNA methods to integrate the heterogeneous data sources. We demonstrate through simulated data and real paired primary and metastatic patient data that the method is effective at resolving cell population dynamics across time points in comparison to more traditional deconvolution methods, providing novel insight into how cell population dynamics can underlie tumor progression.

## 2 Materials and methods

**Fig. 1.**
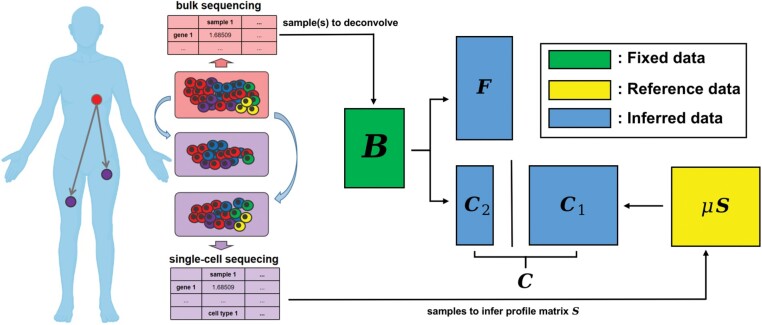
Approach to semi-deconvolution using both bulk and single-cell RNA-seq to uncover the cell type dynamics across progression stages. The left scheme shows an example of tumor sites that might lead to distinct bulk and single-cell RNA-seq samples. The right scheme shows the overall mathematical problem to solve using the bulk and single-cell RNA-seq. B: bulk sample from primary tumor to deconvolve. $C_{1}$: known cell types found in single-cell metastatic samples. $C_{2}$: unknown cell types only in primary tumor. C: horizontal stack of $C_{1}$ and $C_{2}$ to compose the total cell types in primary tumor. F: corresponding fractions of cell types. S: profile matrix inferred from single-cell metastatic samples

Our method can be divided into two steps: First, we infer a profile matrix **S** to represent the single-cell data from metastatic samples. Then, we apply a semi-deconvolution algorithm that uses elements of both partial and complete deconvolution algorithms to avoid weaknesses of each. The objective/loss function consists of two parts: a complete deconvolution part, which by itself would introduce error due to the difficulty of finding a low-dimensional approximation for a noisy high dimensional mixture, and a partial deconvolution part, which by itself would introduce error due to imprecise approximation of the bulk data by single cells gathered from different samples and technologies. By combining these terms, we seek to mitigate the complementary weaknesses of each approach. The approach is particularly effective for characterizing how population frequencies of distinct cell types evolve over progression stages, e.g. via differential immune infiltration (cf. Sturm *et al.*, 2019). Unlike other methods also using reference profiles, our method allows the inference of cell types not found in the single-cell samples. We call our method RAD with single cells (RADs). The operation and a high-level description of the method are summarized in Figure 1.

### 2.1 Mathematical formulation of deconvolution

We aim to achieve better deconvolution by integrating information from the noisy but informative single-cell RNA-seq via the following constrained optimization objective:

(1) $$\underset{C,F,\mu }{\min}\;{\left|\left|B-CF\right|\right|}_{\text{Fr}}^{2}+\lambda ||C_{1}-\mu S|{|}_{\text{Fr}}^{2},$$

 

(2) $$s.t.\;C_{il}\geq 0,\;i=1,\ldots ,m,\;l=1,\ldots ,K,$$

 

(3) $$F_{lj}\geq 0,\;l=1,\ldots ,K,\;j=1,\ldots ,n,$$

 

(4) $$\sum_{l=1}^{K}F_{lj}=1,\;j=1,\ldots ,n,$$

$B\in R_{\geq 0}^{m\times n}$ is the bulk RNA-seq data and $S\in R_{\geq 0}^{m\times k}$ is a preprocessed matrix of single-cell reference data, where each row is a gene, each column of **B** is a bulk sample and each column of **S** is a cell type/population. *λ* is the penalty weight that suppresses the discrepancy between single-cell data and the inferred expression profile matrix and *μ* adjusts the scale of inferred single-cell references. Additional parameters of Equations (1–4) and other auxiliary variables are defined in Table 1.

**Table 1.** Variables and parameters

$B^{m\times n}$	Bulk samples (*m* gene ×*n* samples) in primary tumor
$C_{1}{}^{m\times k}$	Known cell types (*m* gene × *k* known cell types) in primary tumor
$C_{2}{}^{m\times y}$	Possible unknown cell types (*m* gene × *y* unknown cell types) in primary tumor
$F^{K\times n}$	Fraction of cell types (*K* cell types × *n* samples) in primary tumor
$C^{m\times K}$	Total cell types (*m* gene × *K* cell types) in primary tumor; note K=k+y
$C_{S}{}^{m\times k}$	Expression profile (*m* gene × *k* cell types) in metastatic tumor prior to zero-inflation corrections
$S^{m\times k}$	Representative reference (*m* gene × *k* cell types) from single-cell data in metastases
*μ*	Scaling factor for **S**
*λ*	Penalty term to balance information from **S**

### 2.2 Solving the deconvolution problems

The original problem formulation Equations (1–4) is non-negative. Inspired by previous work, we use a coordinate descent method to obtain a possibly suboptimal solution to the problem. The coordinate descent algorithm iteratively repeats three phases described below until convergence, where in each phase, the computational problem can be re-formulated to a tractable problem class, e.g. quadratic programming or linear regression.

*Phase 1: Optimizing fraction matrix* F At this phase, we fix **C** and *μ* to optimize **F**. Then, Equations (1–4) is equivalent to:

(5) $$\underset{F}{\min}\;{\left|\left|B-CF\right|\right|}_{\text{Fr}}^{2},$$

 

(6) $$s.t.\;F_{lj}\geq 0,l=1,\ldots ,k,j=1,\ldots ,n,$$

 

(7) $$\sum_{l=1}^{k}F_{lj}=1,\;j=1,\ldots ,n,$$

Let $f=F_{\cdot j}$ be the column in Equation (5) representing the frequency of each cell type/population in one bulk sample $B_{\cdot j}$ (defined as **b**). Then we can re-formulate it to minimize the error for each column of B−CF:

(8) $$\underset{F}{\min}\;{\left|\left|B-CF\right|\right|}_{\text{Fr}}^{2},$$

 

(9) $$\Leftrightarrow \underset{F}{\min}\;\left[\sum_{j=1}^{n}{\left|\left|B_{\cdot j}-CF_{\cdot j}\right|\right|}_{2}^{2}\right],$$

 

(10) $$\Leftrightarrow \underset{F_{\cdot j}}{\min}\;{\left|\left|B_{\cdot j}-CF_{\cdot j}\right|\right|}_{2}^{2},\;\forall j=1,..,n$$

 

(11) $$\Leftrightarrow \underset{f}{\min}\;{\left|\left|b-Cf\right|\right|}_{2}^{2},(\text{subscript}\;j\;\text{omitted for clarity})$$

 

(12) $$\Leftrightarrow \underset{f}{\min}\;f^{⊺}C^{⊺}Cf-2b^{⊺}Cf+b^{⊺}b,$$

 

(13) $$\Leftrightarrow \underset{f}{\min}\;\frac{1}{2}f^{⊺}C^{⊺}Cf-b^{⊺}Cf$$

Then ${\left(C^{⊺}C\right)}^{⊺}=C^{⊺}{\left(C^{⊺}\right)}^{⊺}=C^{⊺}C$, so $C^{⊺}C$ is symmetric matrix, and further that it will be semi-definite since $f^{⊺}C^{⊺}Cf={\left(Cf\right)}^{⊺}(Cf)\geq 0$, establishing that Equation (13) can be solved as a Quadratic Programming (QP) problem with the same constraints shown in Equations (6) and (7).

*Phase 2: Optimizing cell type matrix* C. At this phase, we fix **F** and *μ* to optimize **C**, then the Equation (1) equals to:

(14) $$\underset{C}{\min}\;{\left|\left|B-CF\right|\right|}_{\text{Fr}}^{2}+\lambda ||C_{1}-\mu S|{|}_{\text{Fr}}^{2},$$

 

(15) $$s.t.\;C_{il}\geq 0,\;\;i=1,\ldots ,m,\;l=1,\ldots ,K,$$

Given that not all primary cell types migrate to metastases, there may be cell types found in the primary tumor but not in single-cell reference samples. We therefore allow the primary tumor to have additional cell types not found in the metastasis, by decomposing **C** into $C_{1}$, representing cell types found in single-cell samples, and an auxiliary matrix $C_{2}$, representing additional cell types present only in bulk samples (Table 1). $C=\left[C_{1},C_{2}\right]$ is the horizontal concatenation of $C_{1}$ and $C_{2}$:

$$\left[\begin{array}{ccc} c_{1,1} & \ldots & c_{1,K}\\ . & \ldots & .\\ . & \ldots & .\\ . & \ldots & .\\ c_{m,1} & \ldots & c_{m,K} \end{array}\right]=\left[\begin{array}{ccccccc} c_{1,1} & \ldots & c_{1,k} & | & c_{1,k+1} & \ldots & c_{1,K}\\ . & \ldots & . & | & . & \ldots & .\\ . & \ldots & . & | & . & \ldots & .\\ . & \ldots & . & | & . & \ldots & .\\ c_{m,1} & \ldots & c_{m,k} & | & c_{m,k+1} & \ldots & c_{m,K} \end{array}\right]$$

Then Equation (14) can be rewritten as:

(16) $$\underset{C_{1},C_{2}}{\min}\;{\left|\left|B-\left[C_{1},C_{2}\right]F\right|\right|}_{\text{Fr}}^{2}+\lambda ||C_{1}-\mu S|{|}_{\text{Fr}}^{2},$$

 

(17) $$s.t.\;{\left(C_{1}\right)}_{il}\geq 0,i=1,\ldots ,m,l=1,\ldots ,k,$$

 

(18) $${\left(C_{2}\right)}_{ij}\geq 0,\;i=1,\ldots ,m,\;j=1,\ldots ,y,$$

We follow coordinate descent to optimize $C_{1}$ and $C_{2}$ iteratively:

*Phase 2.1: Optimizing* C_1_. Let $c_{1}=C_{1}{}_{i\cdot }^{⊺}$, which represents the expression profile of one gene in each cell type/population. Similarly, we define $b=B_{i\cdot }^{⊺},s=S_{i\cdot }^{⊺}$ to represent the transpose of one row of **B** and **S**, respectively. Let $F={\left[F_{1}^{⊺},F_{2}^{⊺}\right]}^{⊺}$ be the vertical concatenation of $F_{1}$ and $F_{2}$. Then we can re-formulate Equation (16) to a standard form for $C_{1}$:

(19) $$\underset{C_{1}}{\min}\;{\left|\left|B-\left[C_{1},C_{2}\right]F\right|\right|}_{\text{Fr}}^{2}+\lambda ||C_{1}-\mu S|{|}_{\text{Fr}}^{2},$$

 

(20) $$\Leftrightarrow \underset{C_{1}}{\min}\;{\left|\left|B-C_{1}F_{1}-C_{2}F_{2}\right|\right|}_{\text{Fr}}^{2}+\lambda ||C_{1}-\mu S|{|}_{\text{Fr}}^{2},$$

 

(21) $$\begin{array}{c} \left(\;\text{let}\;B=B-C_{2}F_{2}\;\text{for convenience}\;\right)\\ \Leftrightarrow \underset{C_{1}}{\min}\;{\left|\left|B-C_{1}F_{1}\right|\right|}_{\text{Fr}}^{2}+\lambda ||C_{1}-\mu S|{|}_{\text{Fr}}^{2}, \end{array}$$

 

(22) $$\Leftrightarrow \underset{C_{1}}{\min}\;\left[\sum_{i=1}^{m}{\left|\left|B_{i\cdot }-{\left(C_{1}\right)}_{i\cdot }F_{1}\right|\right|}_{2}^{2}+\lambda ||{\left(C_{1}\right)}_{i\cdot }-\mu S_{i\cdot }|{|}_{2}^{2}\right],$$

 

(23) $$\begin{array}{c} \Leftrightarrow \underset{{\left(C_{1}\right)}_{i\cdot }}{\min}\;{\left|\left|B_{i\cdot }-{\left(C_{1}\right)}_{i\cdot }F_{1}\right|\right|}_{2}^{2}+\lambda ||{\left(C_{1}\right)}_{i\cdot }-\mu S_{i\cdot }|{|}_{2}^{2},\\ \left(\forall i=1,\ldots ,m,\text{we then omit subscript}\;i\;\text{for clarity}\right) \end{array}$$

 

(24) $$\Leftrightarrow \underset{c_{1}{}^{⊺}}{\min}\;{\left|\left|b^{⊺}-c_{1}{}^{⊺}F_{1}\right|\right|}_{2}^{2}+\lambda {\left|\left|c_{1}{}^{⊺}-\mu s^{⊺}\right|\right|}_{2}^{2},$$

 

(25) $$\Leftrightarrow \underset{c_{1}}{\min}\;\frac{1}{2}c_{1}{}^{⊺}(F_{1}F_{1}^{⊺}+\lambda I)c_{1}-{\left(F_{1}b+\lambda \mu s\right)}^{⊺}c_{1}$$

with the same constraint as shown in Equation (17), where **I** is an identity matrix, which implies $I^{⊺}=I$. Let $Q=F_{1}F_{1}^{⊺}+\lambda I$. We can show that $Q^{⊺}={\left(F_{1}F_{1}^{⊺}+\lambda I\right)}^{⊺}={\left(F_{1}F_{1}^{⊺}\right)}^{⊺}+\lambda I^{⊺}=F_{1}F_{1}^{⊺}+\lambda I=Q$, so **Q** is a symmetric matrix, which indicates that Equation (25) is a QP problem.

*Phase 2.2: Optimizing* $C_{2}\;$. Let $c_{2}=C_{2}{}_{i\cdot }^{⊺}$, which represents the expression profile of one gene in each cell type/population. Similarly, we define $b=B_{i\cdot }^{⊺}$ to represent the transpose of one row of **B**. Then we can re-formulate Equation (16) to a standard form for $C_{2}$:

(26) $$\underset{C_{2}}{\min}\;{\left|\left|B-\left[C_{1},C_{2}\right]F\right|\right|}_{\text{Fr}}^{2}+\lambda ||C_{1}-\mu S|{|}_{\text{Fr}}^{2},$$

 

(27) $$\begin{array}{c} \Leftrightarrow \underset{C_{2}}{\min}\;{\left|\left|B-C_{1}F_{1}-C_{2}F_{2}\right|\right|}_{\text{Fr}}^{2}+\lambda ||C_{1}-\mu S|{|}_{\text{Fr}}^{2},\\ \left(\;\text{let}\;B=B-C_{1}F_{1}\;\text{for convenience}\;\right) \end{array}$$

 

(28) $$\Leftrightarrow \underset{C_{2}}{\min}\;{\left|\left|B-C_{2}F_{2}\right|\right|}_{\text{Fr}}^{2},$$

 

(29) $$\Leftrightarrow \underset{C_{2}}{\min}\;\left[\sum_{i=1}^{m}{\left|\left|B_{i\cdot }-{\left(C_{2}\right)}_{i\cdot }F_{2}\right|\right|}_{2}^{2}\right],$$

 

(30) $$\begin{array}{c} \Leftrightarrow \underset{{\left(C_{2}\right)}_{i\cdot }}{\min}\;{\left|\left|B_{i\cdot }-{\left(C_{2}\right)}_{i\cdot }F_{2}\right|\right|}_{2}^{2},\\ \left(\forall i=1,\ldots ,m,\text{we then omit subscript}\;i\;\text{for clarity}\right) \end{array}$$

 

(31) $$\Leftrightarrow \underset{c_{2}{}^{⊺}}{\min}\;{\left|\left|b^{⊺}-c_{2}{}^{⊺}F_{2}\right|\right|}_{2}^{2},$$

 

(32) $$\Leftrightarrow \underset{c_{2}}{\min}\;\frac{1}{2}c_{2}{}^{⊺}F_{2}F_{2}^{⊺}c_{2}-{\left(F_{2}b\right)}^{⊺}c_{2}$$

We found that Equation (32) has similar form to Equation (13). It is also not hard to see that ${\left(F_{2}F_{2}^{⊺}\right)}^{⊺}=F_{2}F_{2}^{⊺}$, so the term $F_{2}F_{2}^{⊺}$ is symmetric, which also indicates that Equation (32) is a Quadratic Programming problem with the same constraints as Equation (18).

*Phase 3: Optimizing scaling factor* *μ*. At this phase, we fix **F** and $C_{1}$ to optimize *μ*. Then Equation (1) equals to:

(33) $$\underset{\mu }{\min}\;||C_{1}-\mu S|{|}_{\text{Fr}}^{2},$$

Since *μ* is a scalar and $C_{1}$ and **S** are two known matrices with the same dimension of *m *×* l*, then Equation (33) can be viewed as a Linear Regression problem of which the goal is to best fit the model regarding the independent variable **S** to the data $C_{1}$ by using the least-squares method to find the optimal value of *μ* that minimizes the residual sum of squares:

(34) $$\underset{\mu }{\min}RSS(\mu )=\sum_{i}^{m}{\left(C_{1}{}_{i\cdot }-\mu S_{i\cdot }\right)}^{2}=\sum_{i=1}^{m}\sum_{l=1}^{k}{\left(C_{1}{}_{il}-\mu S_{il}\right)}^{2}.$$

In summary, we have shown that the deconvolution problem can be divided into three main phases. In each phase, we have formulated the sub-problem to be either a QP problem or a Linear Regression problem. With third-party software (e.g. CVXOPT), we iteratively solve each phase by using the coordinate descent algorithm until the convergence to get optimal values for $F,C_{1},\;C_{2}$ and *μ*.

### 2.3 Datasets

#### 2.3.1 Simulated datasets

It is not possible to establish with certainty the ground truth for any real deconvolution dataset and so we rely partially on simulated data for validation. Our main strategy is to rely on true single-cell RNA-seq data from paired primary and metastatic breast cancer samples to generate artificial bulk data composed of mixtures of single cells, for which we would then have a known ground truth. We simulated bulk RNA-seq of primary tumor tissue samples based on the following assumptions: (i) the underlying gene expression profile of each cell type in bulk RNA-seq would be similar to the average expression values of cells belonging to the same type in single-cell RNA-seq had dropout events in single-cell RNA-seq not occurred; and (ii) the fractions of different cell types in metastatic tissue samples will be different from those in primary tumor tissue samples.

First, we inferred the cell type expression matrix of bulk RNA-seq from the real single-cell RNA-seq data of metastatic tumor tissue samples. The single-cell RNA-seq results from two distinct metastases of the tumor, BoM1 and BoM2, were normalized to CPM (counts per million) before being aggregated. For each cell type, the aggregated cell expression values were averaged to obtain its gene expression profile. The gene expression profile was normalized to CPM again before being corrected for the effect of single-cell RNA-seq drop-out events (see Section 2.3.3).

The corrected gene expression profile then served as the ground truth cell type expression matrix for simulating the bulk RNA-seq samples. In total, 5000 randomly selected genes and all *k* cell types from the gene expression profile were ${\;\text{log}\;}_{2}$ transformed and replicated *n* times to generate each of the *n* primary tumor tissue sample’s component matrix. Inter-sample noise was added to the component matrices by replacing each of *j*th gene’s expression value *x* with one instance drawn from the Gaussian distribution $N(x,\sigma_{j}/5)$, where *σ_j_* is the standard deviation of *j*th gene’s expression values across cell types in the ${\;\text{log}\;}_{2}$ space. The noisy component matrices were then projected back to the linear space. The fraction vector of the *k* cell types in these primary tumor samples was generated by perturbing the frequency of each cell type in the real single-cell RNA-seq of metastatic tissue samples in ${\;\text{log}\;}_{2}$ scale: each of the *k* frequencies *f_i_* was replaced by one instance drawn from the Gaussian distribution $N(f_{i},\sigma_{f}/2)$, with *σ_f_* being the standard deviation among the *k* cell types’ frequencies. This fraction vector was then replicated *n* times before the addition of inter-sample noise in the same way as the cell type-gene expression component matrix. The fractions for all *k* cell types were then summed to normalize the matrix, resulting in a *n *×* k* fraction matrix where each of the *n* fraction vector sums to 1.

Finally, the *n* simulated bulk RNA-seq of primary tumor tissue samples were generated by convolving each component matrix with its corresponding fraction vector.

#### 2.3.2 Bulk and single-cell RNA-seq datasets

We tested the method on a real dataset consisting of bulk RNA-seq derived from a formalin-fixed parafin embedded primary breast tumor sample and single-cell RNA-seq data derived from two bone metastases. Statistics on the dataset used in the present work can be found in Table 2.

**Table 2.** Matched bulk RNA-seq datasets used in this study

Dataset	PBT	BoM1	BoM2
Data type	Breast primary	Bone metastases at left acetabulum	Bone metastases at right tibia
No. of genes	57 557	18 386	18 386
No. of samples	1	4649	5505
No. of coarse cell types	—	6	6
No. of fine cell types	—	26	24

#### 2.3.3 Preprocessing to construct S

The single-cell RNA-seq experiment produces an $n_{s}\times m$ expression matrix for *m* genes and *n_s_* cells sequenced in total. Using known biomarkers for different cell types, each of the *n_s_* cells could be annotated with one of the *k* cell types identifiable. After normalizing the expression values into CPM, the *k *×* m* matrix $C_{s}$ can be calculated by averaging expression values for each of the *m* genes across cells with the same cell type label.

However, single-cell RNA-seq data includes noise and bias from dropout events, where a gene moderately expressed in some cells failed to be detected in other cells. These drop-outs were hypothesized to be the result of failed reverse transcription during the sequencing experiment, as suggested in previous attempts at quantifying mRNA at single-cell level using RT-qPCR (Bengtsson *et al.*, 2008). These dropout events require additional correction to guide the deconvolution of dropout-free bulk RNA-seq meaningfully. In general, the smaller an expression value is, the higher its probability of being undetected in the experiment will be.

To correct for the effect of dropout events, we assume that the relationship between the *j*th gene’s average expression value $\mu^{j}$ and its dropout frequency follows a Michaelis–Menten function (Andrews and Hemberg, 2019):

(35) $$P_{\text{dropout}}^{j}=1-\frac{\mu^{j}}{K_{M}^{j}+\mu^{j}},$$

where $K_{M}^{j}$ is the Michaelis constant representing the mean expression value of *j*th gene required for half of the cells to be detected. Therefore, we could fit the Michaelis–Menten function with mean expression values and percentages of values dropped from the real single-cell RNA-seq data to estimate the $K_{M}$ for each gene. Then given any average expression value for the cell type, it would be possible to estimate $P_{\text{dropout}}$ using the Michaelis–Menten function. The profile expression vector for the *j*th gene measured in a bulk RNA-seq experiment $S_{j}\in R_{\geq 0}^{1\times k}$ can then be inferred as: $S_{j}=\frac{C_{s}{}^{j}}{1-P_{\text{dropout}}^{j}}$.

### 2.4 Evaluation

We evaluated the performance of our method by comparing the inferred expression profiles $\hat{C}$ and corresponding fractions $\hat{F}$ with the ground truth **C** and **F** by utilizing four different metrics: $R_{C}^{2}$ (Pearson coefficient of $\hat{C}$ and **C**), *L*_1_ loss ($||\hat{C}-C|{|}_{1}/||C|{|}_{1}$), $R_{F}^{2}$ (Pearson coefficient of $\hat{F}$ and **F**) and MSE (mean square error of $\hat{F}$ and **F**). Except where otherwise noted, we used λ=0.1, chosen empirically to give good results across a variety of datasets.

## 3 Results

### 3.1 Our deconvolution is unbiased and robust on simulated data

First, we tested our method on the simulated data generated as discussed in Section 2.3.1. We generated a single-cell matrix **C** with size of *m *=* *5000 random genes and *k *=* *5 known cell types as well as a profile matrix **S** from the current Single-cell RNA (scRNA) dataset. We also allow *y *=* *1 unknown cell type that only exists in the bulk sample. Noise has been introduced to bulk and single-cell samples to mimic the relatively low resolution in bulk sequencing and individual-level differences in single-cell RNA-seq data, respectively.

**Fig. 2.**
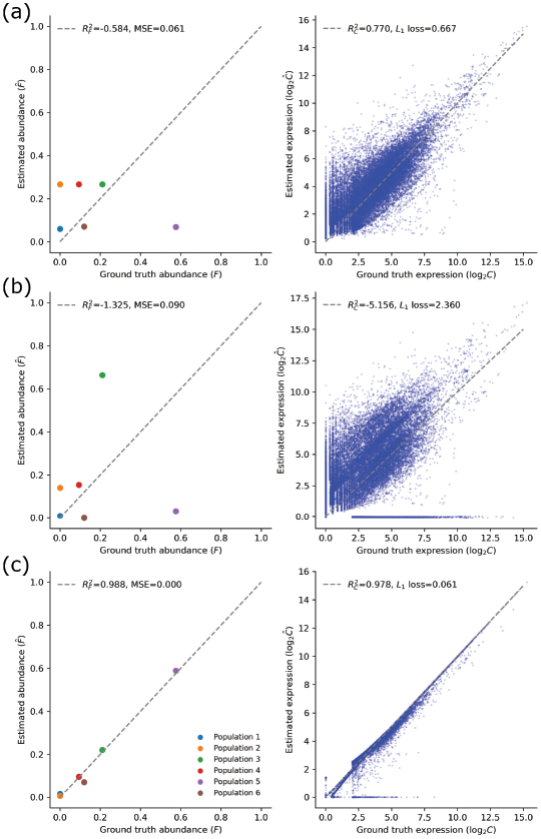
Performance of deconvolution when bulk samples are limited. We show representative results of RAD, RADs without reference, RADs with reference for number of bulk samples *n *=* *1 while number of cell type *K *=* *6 (five known cell types and one unknown cell type). We compared the performance both on estimated C and F using the evaluation metrics *L*_1_ loss, $R_{C}^{2}$ for C and MSE, $R_{F}^{2}$ for F, respectively. (**a**) RAD using S as initialization, (**b**) RADs without S (*λ* = 0) and (**c**) RADs with S (λ=0.1)

When the number of samples increases, the average performance of pure RAD becomes slightly better while the variance is still large. We also find that in most cases, adding a profile matrix **S** further improves the inference by reducing the *L*_1_ loss (Fig. 2a). In the current experiment, we used **S** as the initialization for both RAD and RADs. This is equivalent to feeding prior information to RAD, which is usually not the case for RAD since it assumes that no single-cell information available. Adding such priors could yield faster and better convergence in some cases.

The inference of F shows a similar pattern to that for **C**, although they are not sensitive to small number of samples (Fig. 2b). This is not surprising since **F** has more constraints and the search space of both is much smaller than **C**. Noise was also introduced (b_noise and s_noise are not 0), and we then find that cell component deconvolution with **S** outperforms pure RAD and that of cell component deconvolution without **S** in all cases as well, and it is robust to the noise (Fig. 2). Based on the results, we can conclude that adding information from **C** (e.g. using profile matrix S) can help in bulk sequencing data deconvolution particularly with small numbers of bulk samples.

### 3.2 Comparison with other methods

In this section, we compared our method to two other popular deconvolution methods: DSA, a complete deconvolution method requiring a list of highly expressed genes corresponding to each cell type; and CIBERSORTx, a semi-deconvolution method with reference gene expression profiles from other tissues. There are some restrictions for these methods, e.g. DSA cannot work when there is only one bulk sample to deconvolve and CIBERSORTx requires the number of bulk samples to exceed the number of cell types. In addition, neither of these two methods is able to infer information regarding unknown cell types absent in the reference gene list or expression profiles.

**Fig. 3.**
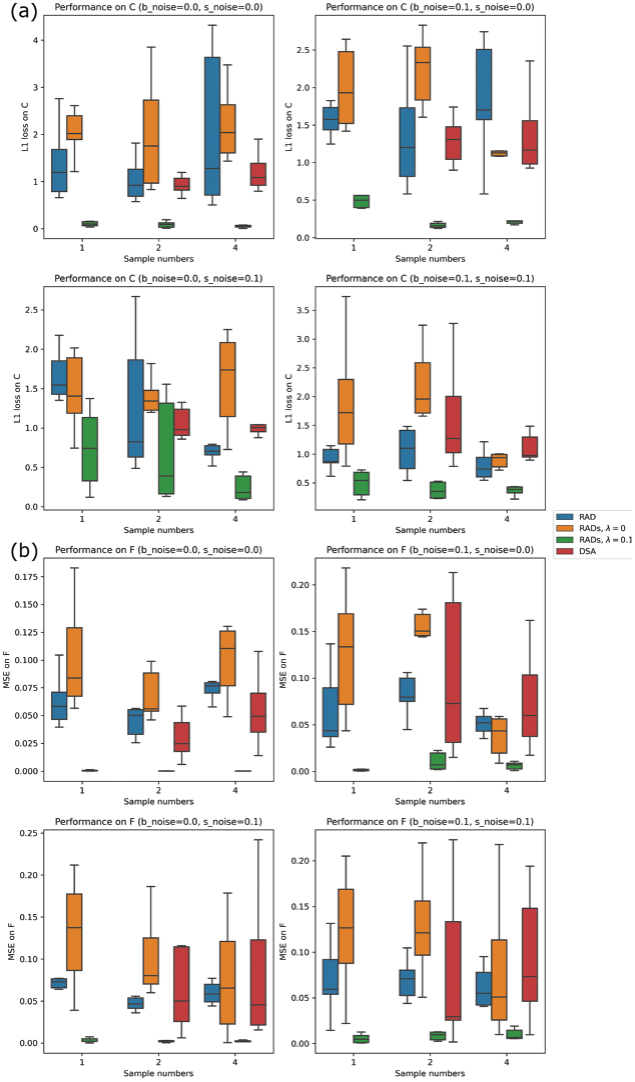
Average performance of deconvolution in various noise (replicates = 10). Average results of RAD, RADs without reference, RADs with reference for number of bulk samples *n *=* *1, 2, 4 and number of cell types *K *=* *6 (five known cell types and one unknown cell type) with different levels of noise (b_noise = 0.0, 0.1, s_noise = 0.0, 0.1). Performance was calculated across 10 replicates, using *L*_1_ loss and MSE for C and F, respectively. (**a**) Performance on C inference, (**b**) Performance on F inference. Different boxes show RAD with S as initialization, RADs without S, RADs with S and DSA, respectively (see Section3.2 for details; note that DSA cannot work when bulk sample *n *=* *1)

In order to make the comparison reasonable, we ran two sets of experiments. In the first experiment, we ran DSA on a small number of bulk samples (e.g. *n *=* *2, 4). Note that DSA can only infer the cell types that are present in **S** so the *L*_1_ loss and MSE were only calculated on the known cell types, although our method includes both known and unknown cell types. We find DSA to have similar performance to RAD and RADs without reference, which is worse than that of RADs with reference in cases with or without noise (Fig. 3, red boxes).

**Fig. 4.**
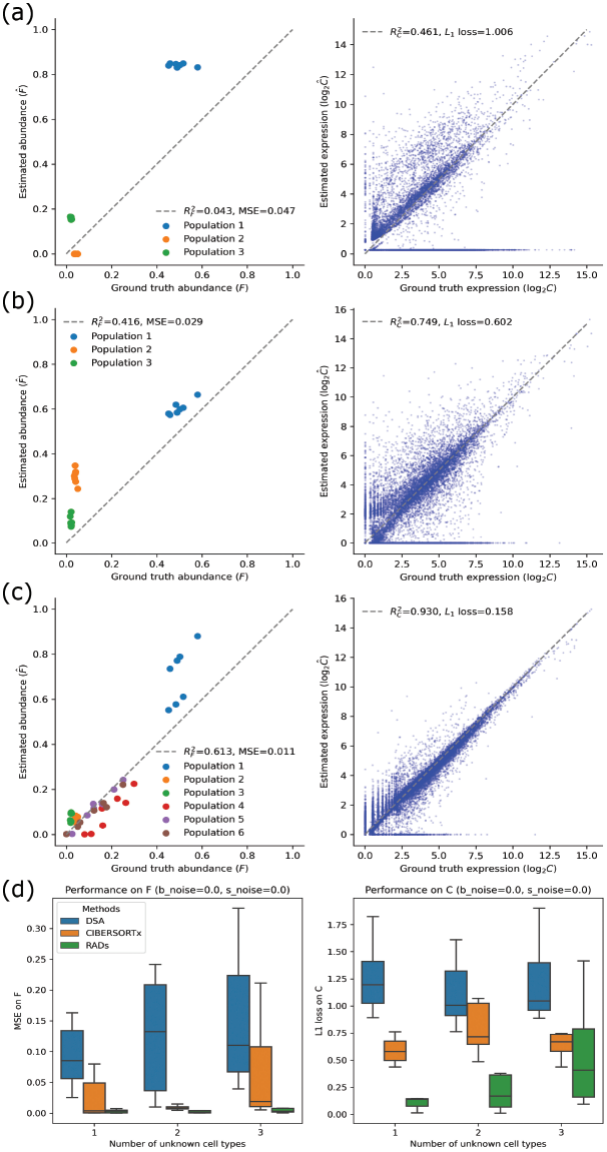
Performance compared with DSA and CIBERSORTx (replicates = 10). Deconvolution on seven simulated bulk samples without noise using six cell types. Unknown cell type(s) were set to be 1, 2 and 3, respectively. (**a–c**) representative result for DSA, CIBERSORTx and RADs when there are three unknown cell types. (**d**) Average performance on F (left) and S (right) inference from the three methods. Metrics of DSA and CIBERSORTx were calculated only on the known cell types in the inferred results and ground truth while sRAD considered both known and unknown cell types

In the second experiment, we increased the number of simulated bulk samples to be 7, but varied the number of known and unknown cell types to be 5:1, 4:2 and 3:3, respectively. This setting is intended to align with requirement of CIBERSORTx and also allow us to investigate the effects of known information on the deconvolution performance. We find that DSA and CIBERSORTx perform worse than our method (Fig. 4d, different boxes). This might be due to the fact that DSA and CIBERSORTx can only infer the cell types in **S** and any unknown cell types then are included in the bulk sample profiles as noise rather than independent components in the deconvolution result, while our method considered both known and unknown cell types in the bulk samples and separated them in the deconvolution result. We also find that when there are more unknown cell types, our method still works but the performance became worse (Fig. 4d), different X labels). This is not surprising since more unknown cell types means less useful information in **S**, which makes the penalty term in Equation (1) less effective. We further note that the problem is ill-posed if the number of unknown cell types exceeds the number of bulk samples. This also indicates the importance of correct reference information in the bulk deconvolution.

### 3.3 Systematic changes in the breast cancer metastatic microenvironment

In this section, we applied our deconvolution method to the real data described in Section 2.3.2. We first retrieved genes that were differentially expressed across different cell types in the single-cell RNA-seq data. For each gene, its ${\;\text{log}\;}_{2}$-scaled expression values in all cells annotated to be one cell type was compared with those in all other cells. A Wilcoxon rank-sum test was performed during the comparison to determine if the gene was significantly differentially expressed in the foreground cell type compared with the background. A Benjamini–Hochberg corrected *P*-value cutoff of 0.001 yielded a total of 1226 genes differentially expressed across all six cell types annotated in the single-cell RNA-seq. Then, we checked with the bulk sample to make sure that the same set of genes is available for both bulk- and single-cell samples, which finally yielded 1196 genes. The expression profiles of the 1196 genes from the bulk sample were then retrieved to compose **B**, and those from single-cell samples were retrieved to compose $C_{S}$. The profile matrix **S** was inferred from $C_{S}$ as described in Section 2.3.3. **B** and **S** are the input for Equation (1). We also allow for one cell type ($C_{2}$) to represent the cell type only found in primary tumor as well to balance the difference between bulk and single-cell sequencing data.

**Fig. 5.**
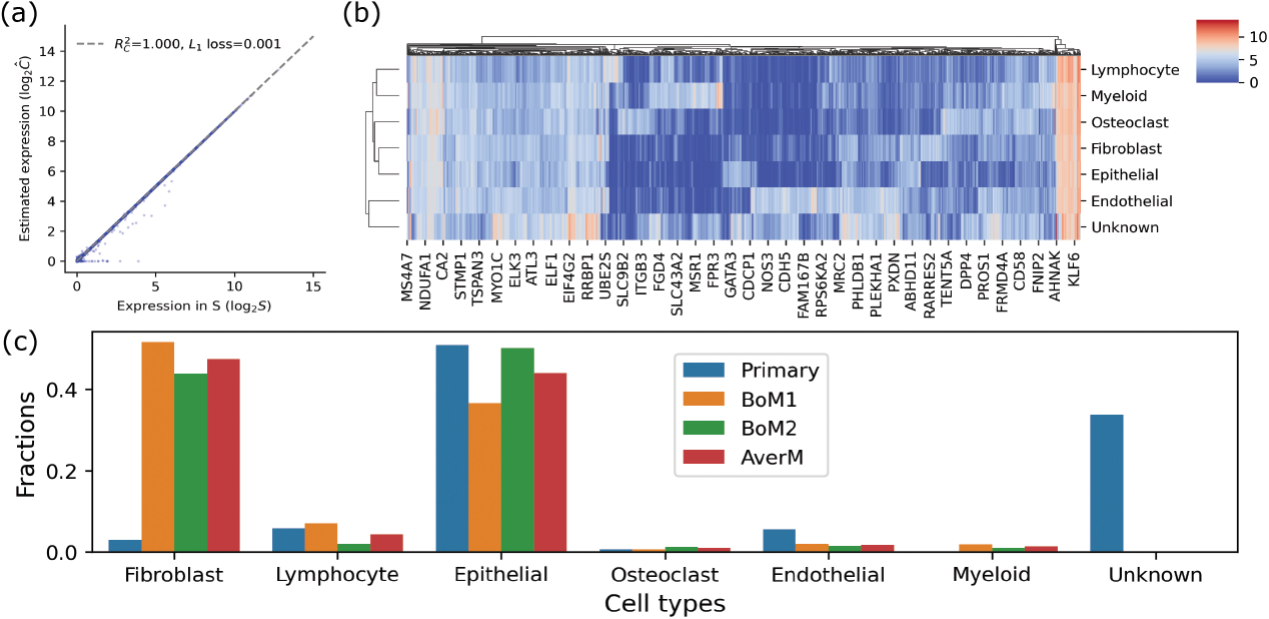
Gene expression in seven cell types and corresponding fractions in primary tumor and bone metastasis. (**a**) Comparison between inferred C and profile matrix S, (**b**) gene expression profiles in inferred C, (**c**) fractions of different cell types in primary tumor and two bone metastasis (BoM1 and BoM2). *AverM* means the average fraction in BoM1 and BoM2

The inferred gene expression profiles from bulk samples were found to match very well with the profile matrix **S** from single-cell samples. This indicates that the penalty term in Equation (1) works with a non-zero regularization term (Fig. 5a, λ=0.1) in real data. The heat-map showing the gene expression also yields distinct expression patterns for different cell types (Fig. 5b). However, fractions of cell types exhibit different patterns between primary site and metastatic sites. For example, the fibroblasts cell type takes a low proportion in the primary site, but makes up for a large one in the metastatic sites. Although the only bulk sample available might have an unrepresentative low fibroblast content at the specific location where the tissue was sampled (e.g. high fibroblasts may be needed for the tumor to attach to the bone), the large proportion of fibroblasts in the bone metastases is consistent with previous studies claiming that cancer-associated fibroblasts contribute to tumor growth, invasion and metastasis (Joshi *et al.*, 2021; Kalluri and Zeisberg, 2006), which leads to cancer malignancy in later stages. The fraction of lymphocytes also exhibits an interesting pattern. The primary sample shows a higher fraction than the average fraction in bone metastatic samples (Fig. 5, rightmost versus leftmost bar) although BoM1 includes more lymphocytes than the primary sample, whereas BoM2 includes fewer. However, the average of BoM1 and BoM2 shows a lower fraction than the primary sample (Fig. 5, leftmost bar). We also find that there are almost no myeloid cells in the primary tumor (fraction = $1.3\times 10^{-4}$) but some in bone metastases. A closer examination found that among the single-cell samples labeled as myeloid cell, macrophages occupy a large proportion (77.4%, from a finer cell-type annotation on the same dataset, data not shown). All these findings are consistent with prior work, which concluded that metastatic breast cancers show reduced immune cells but increased macrophages compared with primary tumors (Zhu *et al.*, 2019). We also found epithelial cells relatively preserved in both primary and metastatic tumors. This is consistent with breast cancer origin from non-diseased epithelial tissue and transition to metastasis (Nguyen *et al.*, 2018; Wang and Zhou, 2011). The fractions of osteoclasts can be used as negative controls since they are bone-tissue related cell types that should be rare in primary sites, and indeed they exhibit fractions close to zero in the primary tumor. The unknown cell types, meaning inferred cell types not found in the single-cell data, account for over 30% in the primary tumor. Although this unknown type is close to endothelial (Fig. 5c) based on the expression distance, we would not assign any biological meaning to it until we have better evidence (e.g. comparing to single-cell data from the same primary tumor or other reliable public data). We interpret it as a free component that accounts for the difference between primary and metastatic tumor composition.

**Fig. 6.**
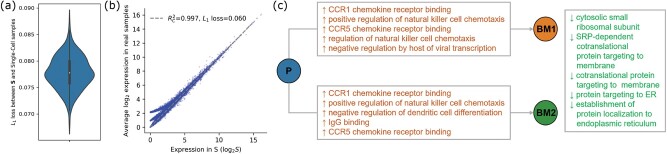
Gene expression in S and real samples and the GO enrichment pathway in primary and metastases (**a**) Violin plot for *L*_1_ loss of profile matrix S and single-cell samples, (**b**) correlation between S and average gene expression profiles (${\;\text{log}\;}_{2}$ scale) in single-cell samples, (**c**) Top GO enrichment pathway changes in primary (P) and two bone metastases (BM1 and BM2) with *α *= 0.05 for Holm-corrected *P* values. Text preceded by up arrows indicates up-regulated pathways whereas text preceded by down arrows indicates down-regulated pathways

We then explored the inferences from the perspective of variations in inferred single-cell gene expression and their interpretation with respect to Gene Ontology (GO) enrichment pathways. We first show that **S** extracted from real data is a good representative of the true single-cell data. It has low average distance to the expression profiles of each of the single-cell samples and shows high correlation with such gene expression profiles (Fig. 6a and b). Although **S** represents the available single-cell samples well while still allowing us to infer a reasonable **C** at the cell-type level, some variations in gene expression were observed between inferred primary tumor **C** and the single-cell RNA-seq data measured from each of the two bone metastases. This may suggest changes in gene expression at the single-cell level along the metastatic trajectory of the tumor. The top 1% most down-regulated and up-regulated genes in each metastatic sample, measured by their distances from the inferred expression values for the primary tumor sample, were selected for downstream GO enrichment analysis (Fig. 6c). The top up-regulated genes in both metastatic samples showed significant enrichment for the receptor binding of several chemokines that have been found to have a direct impact on metastasis, including CCR5 and CCR1 (Mollica Poeta *et al.*, 2019). This is consistent with previous reports that increased in CCR5 activity leads to increased homing behavior to metastatic sites in breast cancer (Jiao *et al.*, 2021). Similarly, the knock-down of CCR1 was experimentally shown to inhibit metastasis of breast cancer (Shin *et al.*, 2017). The up-regulation in both metastatic samples could also have the effect of promoting chemotaxis of natural killer cells that may be recruited in response to increased activity of chemokines CCR1 and CCR5 (Aldinucci *et al.*, 2020). Additionally, increased expression of genes enriched for binding of IgG, such as is observed with BoM2, has been hypothesized to be a driver of breast tumor metastasis (Cui *et al.*, 2021).

## 4 Discussion

In this work, we develop a novel tumor deconvolution method, RADs, designed for scenarios in which we have mixtures of bulk and single-cell data. We show that the method improves on standard bulk deconvolution or reference-based variants and works well even when there are limited numbers of bulk samples. Although our formulation of deconvolution using single-cell data from the same patient alleviates some bias induced by independent single-cell reference profiles, the formulation still implicitly requires all potential populations be available in the single-cell data. The extension of **C** to unknown populations not available in single-cell data can eliminate another source of inaccuracy in reference-based deconvolution. This heuristic idea not only has a biological meaning, such that some cell types in the primary tumor might not migrate to other organs or tissues, but also lends itself well to the coordinate descent algorithm. Unlike other methods, our method directly takes advantage of single-cell data from metastatic sites of the same patient, providing a more accurate reference than those from unrelated normal tissue or a panel of reference samples. In addition, our method can work on a very limited number of bulk samples and still achieve reasonable results when the number of inferred cell types exceeds the number of bulk samples, normally a hard problem for complete deconvolution. The results on the real data align well with existing work on the breast cancer bone metastases as described in Section 3.3.

There are some limitations of our method, however. The method is still limited in its ability to infer new cell types with few bulk samples, making it difficult to characterize progression via novel evolution or complete loss of clones. It would benefit from further evaluation on this point and on its ability to discriminate similar cell types with finer resolution. It additionally depends on some model parameters for which we so far lack principled methods for automated selection. Also, our method requires matched bulk and single-cell samples from the same patient, even if they can come from different sites and progression stages. Although this is motivated by an important use case, the combination of data is still not common. Future work will explore how same-patient and third-party reference data may be synergistic in bypassing this limitation. Nevertheless, we believe our method builds a bridge between limited data and good deconvolution performance, and provides strategies for better leveraging heterogeneous data modalities that may have broader applications in cancer research and other single-cell biology.

## Funding

This work was partially supported by National Institutes of Health awards [R21CA216452 and R01HG010589]; Pennsylvania Department of Health award [No. 4100070287]; Susan G. Komen for the Cure; the Mario Lemieux Foundation; and the Breast Cancer Alliance. It was also partially supported by the AWS Machine Learning Research Awards granted to J.M. and R.S. and by the Center for Machine Learning and Health Fellowship granted to Y.T. The Pennsylvania Department of Health specifically disclaims responsibility for any analyses, interpretations or conclusions.

*Conflict of Interest*: none declared.

## Data availability

Simulated data created for this study and additional results are provided with the RADs source code at https://github.com/CMUSchwartzLab/RADs. The true tumor data, on which the simulations are also based, are being released via the Gene Expression Omnibus (GEO) under accession GSE190772.

## References

[btac262-B1] Aldinucci D. et al (2020) The CCL5/CCR5 axis in cancer progression. Cancers (Basel), 12, 1765. 32630699 10.3390/cancers12071765 PMC7407580

[btac262-B2] Andrews,T.S. and Hemberg,M. (2019) M3Drop: dropout-based feature selection for scRNASeq. *Bioinformatics *, 35, 2865–2867. 30590489 10.1093/bioinformatics/bty1044 PMC6691329

[btac262-B3] Avila Cobos F. et al (2020) Benchmarking of cell type deconvolution pipelines for transcriptomics data. Nat. Commun., 11, 5650. 33159064 10.1038/s41467-020-19015-1 PMC7648640

[btac262-B4] Beerenwinkel N. et al (2016) Computational cancer biology: an evolutionary perspective. PLoS Comput. Biol., 12, e1004717. 26845763 10.1371/journal.pcbi.1004717 PMC4742235

[btac262-B5] Bengtsson M. et al (2008) Quantification of mRNA in single cells and modelling of RT-qPCR induced noise. BMC Mol. Biol., 9, 63. 18631407 10.1186/1471-2199-9-63 PMC2483285

[btac262-B6] Chang K. et al (2013) The cancer genome atlas pan-cancer analysis project. Nat. Genet., 45, 1113–1120. 24071849 10.1038/ng.2764 PMC3919969

[btac262-B7] Cui M. et al (2021) Immunoglobulin expression in cancer cells and its critical roles in tumorigenesis. Front. Immunol., 12, 613530. 33841396 10.3389/fimmu.2021.613530 PMC8024581

[btac262-B8] Greaves M. , Maley C.C. (2012) Clonal evolution in cancer. Nature, 481, 306–313. 22258609 10.1038/nature10762 PMC3367003

[btac262-B9] Jiao X. et al (2021) Leronlimab, a humanized monoclonal antibody to CCR5, blocks breast cancer cellular metastasis and enhances cell death induced by DNA damaging chemotherapy. Breast Cancer Res., 23, 11. 33485378 10.1186/s13058-021-01391-1 PMC7825185

[btac262-B10] Joshi R.S. et al (2021) The role of cancer-associated fibroblasts in tumor progression. Cancers, 13, 1399. 33808627 10.3390/cancers13061399 PMC8003545

[btac262-B11] Kalluri R. , Zeisberg M. (2006) Fibroblasts in cancer. Nat. Rev. Cancer, 6, 392–401. 16572188 10.1038/nrc1877

[btac262-B12] Kuipers J. et al (2017) Advances in understanding tumour evolution through single-cell sequencing. Biochim. Biophys. Acta. Rev. Cancer, 1867, 127–138. 28193548 10.1016/j.bbcan.2017.02.001 PMC5813714

[btac262-B13] Lei H. et al (2020) Tumor copy number deconvolution integrating bulk and single-cell sequencing data. J. Comput. Biol., 27, 565–598. 32181683 10.1089/cmb.2019.0302 PMC7185355

[btac262-B14] Lim B. et al (2020) Advancing cancer research and medicine with single-cell genomics. Cancer Cell, 37, 456–470. 32289270 10.1016/j.ccell.2020.03.008 PMC7899145

[btac262-B15] Lu P. et al (2003) Expression deconvolution: a reinterpretation of DNA microarray data reveals dynamic changes in cell populations. Proc. Natl. Acad. Sci. USA, 100, 10370–10375. 12934019 10.1073/pnas.1832361100 PMC193568

[btac262-B16] Malikic S. et al (2019a) Integrative inference of subclonal tumour evolution from single-cell and bulk sequencing data. Nat. Commun., 10, 1–12. 31227714 10.1038/s41467-019-10737-5 PMC6588593

[btac262-B17] Malikic S. et al (2019b) PhiSCS: a combinatorial approach for subperfect tumor phylogeny reconstruction via integrative use of single-cell and bulk sequencing data. Genome Res., 29, 1860–1877. 31628256 10.1101/gr.234435.118 PMC6836735

[btac262-B18] McCarthy D.J. et al; HipSci Consortium. (2020) Cardelino: computational integration of somatic clonal substructure and single-cell transcriptomes. Nat. Methods, 17, 414–421. 32203388 10.1038/s41592-020-0766-3

[btac262-B19] Menden K. et al (2020) Deep learning–based cell composition analysis from tissue expression profiles. Sci. Adv., 6, eaba2619. 32832661 10.1126/sciadv.aba2619 PMC7439569

[btac262-B20] Mollica Poeta V. et al (2019) Chemokines and chemokine receptors: new targets for cancer immunotherapy. Front. Immunol., 10, 379. 30894861 10.3389/fimmu.2019.00379 PMC6414456

[btac262-B21] Naxerova K. , Jain R.K. (2015) Using tumour phylogenetics to identify the roots of metastasis in humans. Nat. Rev. Clin. Oncol., 12, 258–272. 25601447 10.1038/nrclinonc.2014.238

[btac262-B22] Newman A.M. et al (2019) Determining cell type abundance and expression from bulk tissues with digital cytometry. Nat. Biotechnol., 37, 773–782. 31061481 10.1038/s41587-019-0114-2 PMC6610714

[btac262-B23] Nguyen Q.H. et al (2018) Profiling human breast epithelial cells using single cell RNA sequencing identifies cell diversity. Nat. Commun., 9, 1–12. 29795293 10.1038/s41467-018-04334-1 PMC5966421

[btac262-B24] Salehi S. et al (2017) ddClone: joint statistical inference of clonal populations from single cell and bulk tumour sequencing data. Genome Biol., 18, 1–18. 28249593 10.1186/s13059-017-1169-3 PMC5333399

[btac262-B25] Schwartz R. , Schäffer A.A. (2017) The evolution of tumour phylogenetics: principles and practice. Nat. Rev. Genet., 18, 213–229. 28190876 10.1038/nrg.2016.170 PMC5886015

[btac262-B26] Schwartz R. , Shackney S.E. (2010) Applying unmixing to gene expression data for tumor phylogeny inference. BMC Bioinformatics, 11, 42. 20089185 10.1186/1471-2105-11-42 PMC2823708

[btac262-B27] Shafighi S.D. et al (2021) Cactus: integrating clonal architecture with genomic clustering and transcriptome profiling of single tumor cells. Genome Med., 13, 1–16. 33761980 10.1186/s13073-021-00842-w PMC7988935

[btac262-B28] Shin S.Y. et al (2017) C-C motif chemokine receptor 1 (CCR1) is a target of the EGF-AKT-mTOR-STAT3 signaling axis in breast cancer cells. Oncotarget, 8, 94591–94605. 29212252 10.18632/oncotarget.21813 PMC5706898

[btac262-B29] Sturm G. et al (2019) Comprehensive evaluation of transcriptome-based cell-type quantification methods for immuno-oncology. Bioinformatics, 35, i436–i445. 31510660 10.1093/bioinformatics/btz363 PMC6612828

[btac262-B30] Tao Y. et al (2020a) Neural network deconvolution method for resolving pathway-level progression of tumor clonal expression programs with application to breast cancer brain metastases. Front. Physiol., 11, 1055. 33013452 10.3389/fphys.2020.01055 PMC7499245

[btac262-B31] Tao Y. et al (2020b) Robust and accurate deconvolution of tumor populations uncovers evolutionary mechanisms of breast cancer metastasis. Bioinformatics, 36, i407–i416. 32657393 10.1093/bioinformatics/btaa396 PMC7355293

[btac262-B32] Wang J. et al (2021) Bayesian estimation of cell type–specific gene expression with prior derived from single-cell data. Genome Res., 31, 1807–1818. 33837133 10.1101/gr.268722.120 PMC8494232

[btac262-B33] Wang Y. , Zhou B.P. (2011) Epithelial-mesenchymal transition in breast cancer progression and metastasis. Chin. J. Cancer, 30, 603–611. 21880181 10.5732/cjc.011.10226 PMC3702729

[btac262-B34] Zaitsev K. et al (2019) Complete deconvolution of cellular mixtures based on linearity of transcriptional signatures. Nat. Commun., 10, 2209. 31101809 10.1038/s41467-019-09990-5 PMC6525259

[btac262-B35] Zhang J. et al (2011) International cancer genome consortium data portal–a one–stop shop for cancer genomics data. Database (Oxford), 2011, bar026. 21930502 10.1093/database/bar026 PMC3263593

[btac262-B36] Zhong Y. et al (2013) Digital sorting of complex tissues for cell type-specific gene expression profiles. BMC Bioinformatics, 14, 89. 23497278 10.1186/1471-2105-14-89 PMC3626856

[btac262-B37] Zhu L. et al (2019) Metastatic breast cancers have reduced immune cell recruitment but harbor increased macrophages relative to their matched primary tumors. J. Immunother. Cancer, 7, 265. 31627744 10.1186/s40425-019-0755-1 PMC6798422

